# A Reproducible Benchmark for Gas Sensor Array Classification: From FE-ELM to ROCKET and TS2I-CNNs

**DOI:** 10.3390/s25206270

**Published:** 2025-10-10

**Authors:** Chang-Hyun Kim, Seung-Hwan Choi, Sanghun Choi, Suwoong Lee

**Affiliations:** 1 Department of Advanced Mobility Components Group, Korea Institute of Industrial Technology, Daegu 42994, Republic of Korea; 2 School of Mechanical Engineering, Kyungpook National University, Daegu 41566, Republic of Korea

**Keywords:** Gas Sensor Array (GSA), time-series classification, Time-Series-to-Image (TS2I)

## Abstract

Classifying low-concentration Gas Sensor Array (GSA) data is hard due to low SNR, sensor heterogeneity, drift, and small samples. We benchmark time-series-to-image (TS2I) CNNs against time-series classifiers, after reproducing a strong FE-ELM baseline under a shared fold manifest. Using the GSA-LC and GSA-FM datasets, we compare FE-ELM, vector baselines, time-series methods, and TS2I-CNNs with 20 × 5 repeated stratified cross-validation (*n* = 100). ROCKET delivers the best accuracy on both datasets and is significantly better than TCN and MiniROCKET (paired tests with Holm–Bonferroni, *p* < 0.05): on GSA-FM, accuracy 0.9721 ± 0.0480 (95% CI [0.9627, 0.9815]) with Macro-F1 0.9757; on GSA-LC, 0.9578 ± 0.0433 (95% CI [0.9493, 0.9663]) with Macro-F1 0.9555. Among image-based models, CNN-RP is the most robust, whereas CNN-GASF lags, especially on GSA-LC. RGB fusion strategies (e.g., with MTF) are dataset-dependent and often inconsistent, and transfer learning with ResNet-18 offers no consistent advantage. Overall, ROCKET ranks first across folds, while CNN-RP is the most reliable TS2I alternative under low-concentration conditions. These results provide a reproducible, fair benchmark for e-nose applications and practical guidance for model selection, while clarifying both the potential and limitations of TS2I.

## 1. Introduction

GSAs, commonly known as e-noses, identify gas type and concentration by recognizing complex response patterns from multiple sensors [1,2,3]. This technology underpins manufacturing process monitoring, environmental sensing, and biosignal analysis. However, in real-world low-concentration settings, classification reliability is undermined by reduced SNR, inter-sensor heterogeneity and non-linearity, long-term drift, and limited training data [4,5].

To address these issues, vector-based classifiers with feature engineering—such as Support Vector Machines (SVM), Random Forest, and Extreme Learning Machines (ELM)—have been widely used [6,7,8,9], with AdaBoost and Logistic Regression as comparative baselines [10]. In particular, Zhao et al.’s FE-ELM constructs multiple feature subsets by downsampling high-dimensional time series, trains independent base ELMs on each subset, and ensembles their outputs [1], effectively mitigating overfitting and showing strong performance on low-concentration gas data.

Meanwhile, recent research in time-series classification has seen the emergence of a new alternative: converting data into 2D images to leverage the powerful visual pattern recognition capabilities of CNNs. TS2I techniques like Recurrence Plot (RP) [11,12], Gramian Angular Field (GAF) [13,14], and Markov Transition Field (MTF) [13,15] encode the temporal dependencies and dynamic characteristics inherent in time-series into the texture or structure of an image. These transformations preserve pairwise relations across the entire length of the original data, offering the potential advantage of learning subtle temporal patterns in low-concentration environments without loss.

The objectives of this study are twofold. First, to rigorously reproduce the performance of the previously proposed FE-ELM under the same public datasets and evaluation protocol to validate its effectiveness. Second, using the reproduced FE-ELM as a bench-mark, to conduct a fair and systematic performance comparison with modern time-series classification models, including the aforementioned time-series-to-image transformation CNNs (e.g., RandOm Convolutional KErnel Transform (ROCKET), TCN) [6,7,8,9,10,11,16,17,18,19]. To this end, this study aims to make the following contributions:

(1) establish and release a reproducible experimental pipeline that standardizes the entire process from data transformation, storage, and indexing to model evaluation [19,20];
(2) establish a fair comparison framework based on a ‘fold manifest’ where all models share the same cross-validation splits [21,22,23];
(3) systematically compare and analyze the effectiveness of single image representations, RGB fusion combining multiple representations, and late fusion strategies [24];
(4) provide a comprehensive benchmark from traditional classifiers to the latest deep learning models to offer insights for practical model selection in this field.

## 2. Methods

**Figure 1:**
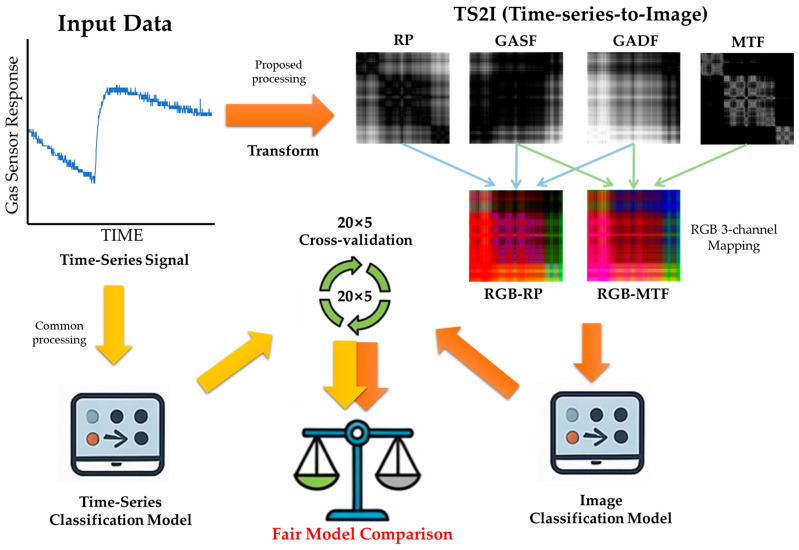
Overview of the TS2I pipeline and benchmark on GSA-LC and GSA-FM. Raw gas-sensor time series are transformed to images via TS2I using RP, GASF/GADF, and MTF. Single-channel outputs can be used directly, or mapped to RGB by channel stacking. In parallel, the same inputs feed sequence models after common preprocessing. All models are trained and evaluated under a shared fold manifest with 20 × 5 repeated stratified cross-validation (*n* = 100) to enable fair, like-for-like comparison. Shown images are representative; intensity ranges are normalized for visualization.

This study designed a modularized pipeline to ensure the reproducibility and scalability of experiments, consisting of data preparation, time-series-to-image transformation, and model training and evaluation stages. Figure 1 provides an at-a-glance overview of our TS2I pipeline, model families, and evaluation design (20 × 5 repeated CV; *n* = 100), together with the headline findings and recommendations.

### 2.1. Datasets and Experimental Design

This research utilized two public benchmark datasets from the UCI Machine Learning Repository to ensure an objective performance evaluation and the reproducibility of our findings. The first dataset, ‘Gas Sensor Array Low-Concentration’ (GSA-LC), consists of time-series data from 10 sensors for 6 types of gases, where each sample has a length of 900 timesteps [1,25]. The second, ‘Gas Sensor Array Flow-Modulated’ (GSA-FM), contains responses from 16 sensors to 4 gases (including air), with each sample comprising 7481 timesteps [1,26,27]. For GSA-LC, each sample comprises 9000 time points formed by concatenating the responses of 10 sensors in fixed order—TGS2603, TGS2630, TGS813, TGS822, MQ-135, MQ-137, MQ-138, 2M012, VOCS-P, 2SH12—while for GSA-FM, the array contains 16 MOX sensors spanning five Figaro TGS models; acquisition/platform details are as documented in the respective sources.

In summary, GSA-LC comprises 90 samples (6 gases (ethanol, acetone, toluene, ethyl acetate, isopropanol, and n-hexane) × 15) and is balanced. GSA-FM comprises 58 samples with four labels (mixture × 20, ethanol × 15, acetone × 15, air × 8) and is imbalanced. All interpretations are confined to within-dataset comparisons.

Model evaluation was conducted using a 20 times 5-fold cross-validation scheme. To guarantee a fair comparison, a shared fold manifest detailing all 100 (20 × 5) training and test splits was pre-generated [22,23,24]. By referencing this manifest, all models were trained and tested under identical data split conditions, thus precluding any performance variations arising from the randomness of data partitioning and ensuring a reliable comparison.

### 2.2. Time-Series to Image Transformation for Feature Extraction

In this study, we adopted an approach that transforms one-dimensional (1D) time-series data measured from each sensor into two-dimensional (2D) image representations before using them as input for classification models. The core idea of this approach is to recast the temporal relationships within a 1D time series into spatial relationships within a 2D image. This process is analogous to converting an audio waveform into a spectrogram, where time-frequency patterns become visible textures that a computer vision model can analyze. This TS2I paradigm has garnered considerable attention in the field of time-series classification over the past decade. The core philosophy of this approach is to encode the complex dynamic patterns and temporal dependencies inherent in 1D time-series into the texture, topology, and geometric structures of a 2D image. This enables the direct application of powerful computer vision models, such as CNNs, which have achieved overwhelming success in natural image recognition, to the analysis of time-series data. CNNs are specialized in extracting local features from an image and learning complex patterns through their hierarchical combination, making them effective at capturing the subtle temporal patterns hidden within TS2I-transformed images.

In this work, we utilized three prominent TS2I techniques to convert each sensor’s time-series data into images: RP, GAF, and MTF. Before TS2I, we apply per-sensor z-score standardization using training-set statistics to mitigate cross-sensor scale differences. This normalization largely harmonizes channel ranges prior to stacking. All generated transformation outputs were saved in two formats: lossless *.npy* arrays with float32 type for precise analysis, and 16-bit PNG images for visualization and distribution compatibility. For training, we use float NPY tensors without display scaling, so grayscale stretching in PNG exports does not affect learning.

TS2I hyperparameters follow the released implementation; we combine code defaults with small validation checks and conventional choices, and we explicitly state the per-transform settings below.

#### 2.2.1. Recurrence Plot

The Recurrence Plot is a technique rooted in dynamical systems theory that visualizes how the state of a system returns to its previous states over time. In other words, it represents how similar the state at a specific time point is to the states at all past time points within a 2D matrix. This method is highly effective for capturing complex dynamic patterns such as the non-linear characteristics, periodicity, and deterministic structure of time-series data.

Transformation Process: The first step to generate an RP is to reconstruct the 1D time-series into a high-dimensional phase space. This is performed using the time-delay embedding technique according to Takens’ Embedding Theorem [28]. Given an original time-series *X* = {*x*_1_, *x*_2_, …, *xn*}, state vectors in the phase space are created using two hyperparameters: an embedding dimension m and a time delay *τ*, such that *v_i_* = (*x_i_*, *x*_*i*+*τ*_, …, *x*_*i*+(*m*−1)*τ*_). Subsequently, the distance between all pairs of state vectors (*v_i_*,*v_j_*) is calculated, and if the distance is smaller than a predefined threshold (*ϵ*), it is considered a recurrence, forming the RP matrix $R_{i,j}$

(1) $$R_{i,j}=\Theta \left(\epsilon -||x_{i}-x_{j}||\right)$$

where $R_{i,j}$ represents the recurrence state between time points i and j, ϵ is the threshold distance, and Θ is the Heaviside step function [29].

In our implementation, RP does not use time-delay embedding parameters (no *m* or *τ*) and is controlled by an RP mode. The default is distance; the gaussian variant auto-estimates *σ* from the data; and the threshold variant sets *ε* to the median similarity. This mode-based RP reduces parameterization and provides stable similarity maps under low SNR.

#### 2.2.2. Gramian Angular Field

GAF is a unique technique that encodes the temporal correlations of a time-series into the topological relations of an image. It holds the theoretical advantage of enabling a bijective mapping, meaning the transformation can be performed without information loss.

Transformation Process: First, the time-series *X* is normalized to a specific range (e.g., [−1, 1] or [0, 1]). Then, each value of the normalized time-series is converted into a polar coordinate system. The value becomes the radius (*r*), and the time index becomes the angle (*ϕ*).

(2) $$\phi_{t}=arccos\left({\overset{∼}{x}}_{i}\right),\;r_{i}=\frac{t_{i}}{N}$$

Here, ${\overset{∼}{x}}_{i}$ is the normalized time-series value, $t_{i}$ is the time stamp, and *N* is a constant factor to regulate the polar coordinate space. Based on this polar representation, the GAF matrix is generated by applying a trigonometric function to the sum or difference in angle pairs. Two variants exist: the Gramian Angular Summation Field (GASF) and the Gramian Angular Difference Field (GADF).

(3) $$G_{i,j}=cos\left(\phi_{i}+\phi_{j}\right),$$

where $\phi_{i}=arccos\left(x_{i}\right)$ represents the angular encoding of the signal [13].

The main diagonal of the GASF matrix preserves the original value information of the time-series and visually represents how correlations change as the temporal distance increases.

This method generates a matrix by transforming a normalized time-series into a po-lar coordinate system and then applying trigonometric functions to the sum (GASF) or difference (GADF) of angle pairs. This transformation encodes temporal correlations into topological relations, preserving them in the image.

GAF applies min–max normalization to [−1, 1] after standardization and uses a simple time-index angle mapping φ = πt/T. This follows common practice and keeps phase coding consistent while remaining easy to reproduce.

#### 2.2.3. Markov Transition Field

MTF approaches time-series from a probabilistic perspective. It quantizes the distribution of time-series values into several bins and represents the transition probabilities from one bin to another as a 2D matrix along the time axis. This is effective for representing the long-term dynamic transition structure of the time-series.

Transformation Process: First, *Q* quantile bins are defined based on the distribution of the time-series values. Each value in the time-series is assigned to its corresponding bin. Then, a *Q × Q* Markov transition matrix *M* is constructed by counting the transitions between bins over all time points. *M_ij_* represents the probability of transitioning from bin *i* to bin *j*. Finally, the MTF is constructed by aligning this transition matrix along the temporal axis. The value at position (*i*, *j*) in the MTF matrix represents the probability of transitioning from the state (bin *q_i_*) at time i to the state (bin *q_j_*) at time *j*, which is *M*_*qi*,*qj*_ [13].

MTF uses *Q =* 64 quantile bins by default and projects first-order transition probabilities along time. This setting mitigates sparsity-related variance and yielded stable fields across datasets.

### 2.3. Model Architectures and Learning Strategies

In this study, three categories of models were comprehensively compared and evaluated for time-series sensor data classification: traditional vector-based models, modern time-series classifiers, and image-based CNNs. The key hyperparameters for each model are summarized in Table 1. For traditional vector-based classifiers, the search space for parameters such as *C*, *gamma*, and *n_estimators* was configured and optimized using the *grid_search_inner_cv* function. Search grids were defined as literature-informed, task-bounded ranges and validated by small validation sweeps; per-fold selections are logged. For clarity, ResNet-18 (transfer) uses ImageNet pretraining with first-conv C adapted to M channel adaptation and a brief freeze–unfreeze schedule, whereas SimpleCNN (from scratch) employs no pretraining, a smaller parameter budget, no freezing, and the same optimizer/scheduler.

#### 2.3.1. Traditional Vector-Based Classifiers

This category includes classical machine learning models that process time-series by transforming them into fixed-size vectors. The core of this approach lies in a preprocessing pipeline that condenses temporal features into a single vector representation.

This category includes widely used classifiers such as SVM for learning non-linear boundaries, Random Forest (RF), which ensembles decision trees, the AdaBoost boosting algorithm, and the Multilayer Perceptron (MLP).

In our work, the common preprocessing pipeline was implemented within the *run_outer_cv_baseline* function.

- Flattening: The 3D time-series data (samples, sensors, time points) was transformed into a 2D matrix (samples, features).
- Standardization: Each feature was standardized to have a zero mean and unit variance (z-score normalization) using *StandardScaler*.
- Dimensionality Reduction: Principal Component Analysis (PCA) was employed to reduce the dimensionality of the feature space.

The classifiers were implemented using their respective classes, such as *svm.SVC* and *ensemble.RandomForestClassifier*.

The main hyperparameters were optimized via grid search. These include the variance ratio to preserve in PCA (*pca_var*), the regularization strength *C* for SVM, the kernel coefficient *gamma* for SVM-RBF (SVM-K), the ensemble size *n_estimators* for RF and AdaBoost, and the number of neighbors *k* for k-NN.

- Random Vector Functional Link (RVFL)
  Model Description: A type of single-hidden-layer neural network that combines the input features with non-linear features generated by a random projection of the input. This augmented feature vector is then trained with a linear classifier.
  Implementation Details: Implemented in the RVFL class, the feature combination is performed using Numpy’s concatenate function. A linear Ridge Regression model serves as the final classifier.
  Key Hyperparameters: The model’s complexity is governed by the number of hidden neurons (hidden) and the choice of activation function (act), which determines the non-linearity.
- Bagging ELM (BAGELM)
  Model Description: An ensemble of ELM trained on bootstrap-resampled subsets of the training data. Each base ELM uses a single hidden layer with randomly initialized hidden weights/biases (kept fixed) to produce a non-linear feature map, and fits output weights via ridge-regularized least squares. Final predictions are obtained by probability averaging (or majority voting) across members, which reduces the variance of a single ELM.
  Implementation Details: Implemented as an ensemble wrapper around an ELM base learner. For each member, a stratified bootstrap sample is drawn; hidden weights/biases are re-initialized; the output layer is solved with ridge regression. At inference, member class probabilities are averaged to yield calibrated predictions; ties fall back to argmax of the averaged scores.
  Key Hyperparameters: M number of ELM members in the ensemble (e.g., 50–200). hidden (L) number of hidden neurons in each ELM (controls feature capacity). hidden-layer activation (e.g., sigmoid/tanh/ReLU). Lambda (λ) ridge regularization strength for the output weights.
- Feature Ensemble ELM
  Model Description: A unique ensemble model that takes 3D time-series as direct input, decomposing the temporal axis into multiple ‘phases’ to generate a diverse set of features. Each feature set is processed by an independent ELM, and the final prediction is determined by a majority vote.
  Implementation Details: This was implemented as the FE-ELM class. The core logic of phase decomposition is handled by the _make_phase_features method.
  Key Hyperparameters: The model’s structure is primarily defined by the number of phases (tau), which controls the ensemble size and the granularity of feature extraction, and the number of hidden neurons in each ELM (hidden).

#### 2.3.2. Modern Time-Series Classifiers

This category comprises state-of-the-art algorithms specifically designed to effectively learn the temporal characteristics of time-series data. The key hyperparameters and default settings for the modern classifiers benchmarked in this study are summarized in Table 1.

- ROCKET/MiniROCKET
  Model Description: These models use 1D convolutions to extract robust features from time-series with exceptional speed. ROCKET generates thousands of random kernels, while MiniROCKET combines a fixed kernel with random parameters.
  Implementation Details: Implemented as the RocketLite and MiniRocketLite classes, respectively. Both generate feature vectors via their transform methods, which are ultimately classified by Scikit-learn’s linear_model.RidgeClassifierCV.
  Key Hyperparameters: The dimensionality of the transformed feature space is determined by the number of kernels (n_kernels) for ROCKET, and the number of features to generate (n_features) for MiniROCKET.
- Temporal Convolutional Network (TCN)
  Model Description: A deep learning architecture for learning long-term dependencies in time-series. It uses dilated causal convolutions to efficiently process wide temporal ranges and employs residual connections for stable training of deep networks.
  Implementation Details: Implemented as a TCN class inheriting from torch.nn.Module within the PyTorch 1.12.1 framework. Its core module, TemporalBlock, is composed of layers such as *nn.Conv1d* and *nn.BatchNorm1d*.
  Key Hyperparameters: The network architecture is defined by the number of TemporalBlocks (tcn_levels) and hidden channels (tcn_hidden). The training process is controlled by parameters such as the learning rate (lr), batch_size, maximum epochs, and early stopping patience.

#### 2.3.3. Image-Based CNNs

The most innovative approach transforms 1D time-series data into 2D images, leveraging CNNs that are highly optimized for image recognition. Fundamentally, a CNN operates by sliding small filters across an image to detect local features such as edges and textures. As data passes through the network’s layers, these simple features are progressively combined to learn more complex and abstract patterns relevant to the classification task. This methodology analyzes temporal patterns by converting them into visual textures and shapes, holding the potential to learn complex relationships that are difficult to capture with other methods. For multivariate time-series, we employed a Sensor Stack strategy, where data from M sensors are first converted into individual 2D images and then stacked as channels of a single multi-channel image.

This pipeline was implemented using a custom *torch.utils.data.Dataset* class. This class reads the M image files composing a single sample and stacks them along the channel axis using Numpy’s concatenate function, creating a final tensor of shape (M, H, W). A helper function, *_adapt_first_conv*, dynamically modifies the first convolutional layer of these models to accept M input channels instead of the standard 3, enabling transfer learning on the Sensor Stack data.

- Implementation Strategies

1. Single Representation (Sensor Stack)

This strategy, which was the focus of the implementation in this study, involves generating 2D images from each sensor using a single transformation technique (e.g., RP). For multi-sensor data, these images are stacked along the channel axis to form a “Sensor Stack” tensor. This creates a multi-channel image with M sensor inputs, analogous to how a color image consists of R, G, and B channels, allowing the CNN to learn inter-sensor interactions simultaneously.

2. RGB Fusion

This method involves normalizing the results of three different transformation techniques (e.g., GADF, GASF, MTF) and assigning them to the R, G, and B channels of a single image. The goal is to induce complementary feature learning by fusing information from multiple representations.

3. Late Fusion

This is an ensemble technique where the probability vectors predicted by multiple independent CNN models, each trained on a different image representation (e.g., an RP-based CNN and a GADF-based CNN), are arithmetically averaged to determine the final class.

Note. Throughout this paper, “RGB” denotes a 3-channel mapping of three transformations to R/G/B; it is not a 4-stack. The per-sensor “Sensor Stack” uses M-channel inputs independently of RGB fusion.

- Detailed Implementation of Sensor Stack

Data Pipeline The dataset was constructed based on an index.csv file that maps each sample to its corresponding sensor image paths. The custom *SensorStackDataset* class performs the following key roles:

Image Loading: It reads the M sensor images for each sample, prioritizing .npy files for processing speed and falling back to .png files when necessary.

Tensor Construction: It stacks the M loaded images along the channel axis to form a single multi-channel input tensor of shape (M, H, W). We evaluated post-stack cross-sensor normalization in an ablation and found no statistically significant gains (differences within CIs). We therefore retain per-sensor z-score as the default for simplicity and reproducibility.

Preprocessing: All images are resized to a specified dimension (e.g., 224 × 224) using bilinear interpolation, and simple data augmentation, such as horizontal and vertical flips, can be applied during training.

- Model Architecture and Transfer Learning

Transfer Learning Strategy:

Dynamic Input Layer Adaptation: Since pre-trained models assume a standard 3-channel (RGB) input, the first convolutional layer was dynamically modified to accept the M-channel “Sensor Stack” data.

Weight Initialization: To preserve the benefits of pre-training, the weights of the new input layer were initialized with the average of the original pre-trained 3-channel weights. This is an effective technique for retaining learned low-level features (e.g., edge and texture detection).

Freeze Backbone: During transfer learning, the weights of the model’s backbone (all layers except the final classifier) could be frozen to allow for rapid and stable fine-tuning on smaller datasets.

#### 2.3.4. Hyperparameter Optimization and Evaluation Protocol

A rigorous and systematic approach was taken to optimize the hyperparameters for every model to ensure that each performed at its peak capability, thereby providing a fair basis for comparison. We report fold-wise CIs and perform paired comparisons consistent with variance-aware inference for cross-validation (e.g., corrected resampled tests), following Nadeau and Bengio’s analysis of the CV estimator’s variance [23].

Optimization of Vector-based Models For the traditional vector-based classifiers, an extensive Grid Search was performed within a nested cross-validation framework. The outer loop was used for performance evaluation, while a dedicated inner 5-fold cross-validation loop was used exclusively for hyperparameter tuning on each training set. To mitigate distributional effects, we used repeated stratified 5-Fold splits and reported macro-F1 alongside accuracy. Statistical significance was assessed on fold-wise scores (CIs and pairwise tests), which controls interpretability concerns due to class skew. This meticulous process ensures that the model selection is unbiased by the test data. The search space was comprehensive; for instance, the regularization parameter C for SVM was searched over a wide range (e.g., 10^−5^ to 10^2^), and the number of estimators for Random Forest was varied (e.g., 200 to 800).

Optimization of Deep Learning Models (TCN and CNNs) The optimization of deep learning models involved a systematic experimental process. Key architectural choices—such as the specific backbone, the use of pre-trained ImageNet weights, and the strategy of freezing backbone layers—were evaluated. Training hyperparameters, including the learning rate, batch size, and weight decay, were carefully configured. Crucially, Early Stopping was employed during training; the model’s performance was monitored on a validation set after each epoch, and training was halted when performance ceased to improve, which implicitly determines the optimal number of training epochs.

Performance Evaluation and Statistical Testing. We adopt 20 × 5 repeated stratified cross-validation (20 repeats × 5 folds), yielding *n* = 100 paired observations per comparison under a shared fold manifest. We report mean accuracy ± std, 95% CIs for the mean, and Macro-F1. Pairwise comparisons use paired *t*-tests on accuracy, reporting Δ(Acc) = A − B, 95% CI for Δ (*t*_0.975_, *df* = 99), two-sided *p*, and Holm–Bonferroni adjusted *p* within pre-specified families (transform/fusion; transfer learning). Effect sizes are given as Cohen’s $d_{z}=t/\sqrt{n}$. Accuracy is used for hypothesis testing; Macro-F1 complements class-imbalance considerations.

## 3. Results

This study evaluated model performance on two public Gas Sensor Array datasets, GSA-FM and GSA-LC, using a 20 × 5 repeated cross-validation scheme. All comparisons were based on 100 paired observations under a shared fold manifest to ensure fairness.

### 3.1. Overall Model Performance

Table 2 summarizes the performance of all evaluated models on GSA-FM and GSA-LC under the shared 20 × 5 repeated stratified CV protocol (*n* = 100 paired observations). We report Macro-F1 as the primary metric, together with Accuracy (mean ± std) and 95% CIs computed from fold-wise scores. On GSA-FM, ROCKET attains the top result (Macro-F1 = 0.9757; Accuracy = 0.9721 ± 0.0480, 95% CI [0.9627, 0.9815]), followed closely by FE-ELM (Macro-F1 = 0.9679). TCN and CNN-RP are competitive (≈0.95 Macro-F1) but are significantly below ROCKET according to the paired, variance-aware CV tests. Early RGB fusion with MTF underperforms relative to single-transform baselines, whereas late fusion (RP + GADF) recovers part of the gap. On GSA-LC, ROCKET again ranks first (Macro-F1 = 0.9555; Accuracy = 0.9578 ± 0.0433, 95% CI [0.9493, 0.9663]), with FE-ELM next (Macro-F1 = 0.9407). MiniROCKET is mid-pack, and within the image models CNN-RP is the most reliable, while CNN-GASF and RGB (GADF, GASF, MTF) lag behind. Across both datasets, transfer learning with ResNet18 does not provide a systematic advantage over SimpleCNN, consistent with the domain-mismatch discussion.

**Figure 2:**
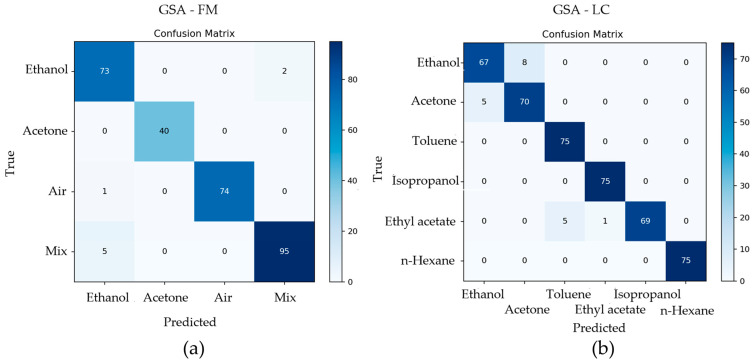
Confusion matrices for the best-performing setting. (**a**) GSA-FM and (**b**) GSA-LC under the shared 20 × 5 repeated stratified cross-validation (*n* = 100). Cell values denote aggregated test predictions per class across folds; darker color indicates higher counts. Class order matches the axis labels in each panel. This side-by-side layout enables a direct comparison of class-wise errors between datasets.

Across both datasets, ROCKET achieved the highest classification accuracy. On GSA-FM, it reached 0.9721 ± 0.0480, and on GSA-LC it attained 0.9578 ± 0.0433. The con-fusion matrices in Figure 2—panels (a) GSA-FM and (b) GSA-LC—provide a class-level view of ROCKET’s predictions, showing minimal misclassifications on both datasets. The reproduced FE-ELM followed closely, confirming its role as a strong baseline. Detailed metrics for all models, including mean accuracy, 95% confidence intervals, and Macro-F1 scores, are reported in Appendix A Table A1 and Table A2.

Conversely, it is noteworthy that traditional vector-based models such as MLP, ELM, and RVFL consistently ranked among the lowest-performing classifiers on both datasets. This pattern suggests that these simpler architectures have limitations in capturing the complex temporal dynamics inherent in GSA time-series, thereby highlighting the advantages of specialized models like ROCKET and FE-ELM.

**Figure 3:**
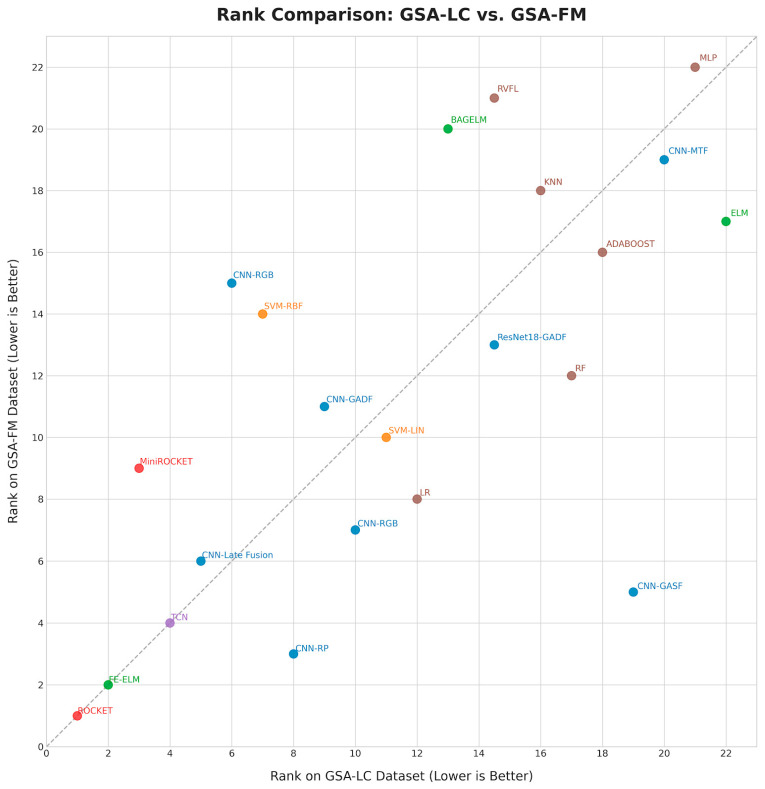
Critical difference rank diagram. Rank comparison between datasets (x-axis: GSA-LC, y-axis: GSA-FM; lower is better). The dashed y = x line indicates agreement in ordering; deviations indicate dataset-specific shifts.

Statistical tests confirmed that ROCKET’s performance was significantly superior to both TCN and MiniROCKET across both datasets (*p* < 0.001). While ROCKET was also significantly better than FE-ELM on the GSA-LC dataset (*p* = 0.00163), the performance difference on the GSA-FM dataset was not statistically significant (*p* = 0.274). An additional noteworthy finding from the pairwise tests is the statistical equivalence in performance between FE-ELM and MiniROCKET on the GSA-LC dataset (*p* = 0.550). This result is intriguing, as it suggests that a specialized feature ensemble method can achieve performance comparable to a lightweight, kernel-based approach like MiniROCKET on this specific task. Detailed statistics for these pairwise comparisons are available in Table 3 and Table 4. The consistent top-tier performance of ROCKET, FE-ELM, and CNNs based on Recurrence Plots (CNN-RP) is also visualized in the critical difference diagram in Figure 3. Figure 3 compares per-dataset ranks (x: GSA-LC, y: GSA-FM, lower is better) with a y = x diagonal marking agreement. ROCKET lies at (1, 1) and FE-ELM near (2, 2), indicating stable ordering across datasets; SimpleCNN is similarly close to the diagonal. CNN-RP falls below the diagonal near (8, 3), reflecting a relatively better standing on GSA-FM, while CNN-GASF is pushed far to the right on GSA-LC (weaker on LC) yet mid-pack on GSA-FM, echoing our per-dataset tables. MiniROCKET sits above the diagonal near (3, 9), performing comparatively better on GSA-LC. Overall, large deviations from the diagonal identify methods with dataset-specific sensitivity, particularly early RGB with MTF, whereas methods on the diagonal (ROCKET, ELM) generalize their relative rank across datasets.

### 3.2. Analysis of Time-Series-to-Image and Fusion Strategies

Among the image-based CNN models, the Recurrence Plot (RP) transformation proved to be the most robust and high-performing method. As summarized in Table 5, On the GSA-LC dataset, CNN-RP was decisively superior to CNN-GASF (ΔAcc = +0.1200, *p* < 1 × 10^−15^), and on the GSA-FM dataset, it significantly outperformed CNN-GADF (ΔAcc = +0.0891, *p* < 10^−11^). In contrast, the GASF transform showed unstable performance that was highly sensitive to the dataset. This instability is highlighted by the statistical results: while its performance was statistically indistinguishable from the top-performing CNN-RP on the GSA-FM dataset (*p* = 0.381), it was massively outperformed on the GSA-LC dataset (*p* < 10^−15^).

Fusion strategies that combine different transformations had limited success. The impact of early fusion strategies, where different transformations are stacked as RGB channels, was highly dataset-dependent. Specifically, including MTF was detrimental on the GSA-FM dataset, significantly degrading performance. In stark contrast, the MTF-inclusive model achieved the highest accuracy among all image-based methods on the GSA-LC dataset. Late fusion, which averages the prediction probabilities of independent models (RP + GADF), was competitive but did not consistently outperform the best single model, CNN-RP.

To contextualize the performance of these TS2I approaches, they were also directly compared against the strong FE-ELM baseline. The results, detailed in Table 6, reveal that while several image-based models significantly underperformed, the top-performing CNN-RP was statistically competitive. For instance, on the GSA-LC dataset, CNN-GASF was significantly worse than FE-ELM (*p* < 10^−7^), but the performance gap between CNN-RP and FE-ELM was not statistically significant. This indicates that while not universally superior, the best TS2I-CNN approach achieves a level of performance comparable to a specialized, high-performing time-series ensemble model.

### 3.3. Efficacy of Transfer Learning

Utilizing a pre-trained ResNet18 model for transfer learning did not provide a consistent advantage on these datasets. As shown in Table 7, for the RP transform on GSA-FM and the GADF transform on GSA-LC, the ResNet18-based model performed significantly worse than a simpler, custom CNN architecture (*p* < 0.05).

## 4. Discussion

The results of this study offer several key insights into model selection for classifying low-concentration gas-sensor data under a reproducible evaluation protocol. By holding the fold manifest fixed across families of methods, we were able to attribute observed performance differences to modeling choices rather than to variability in data partitioning.

First, the superior performance of ROCKET suggests that large, randomized convolutional feature banks are exceptionally effective at extracting discriminative temporal patterns without incurring the overfitting risks associated with deeper, highly parameterized architectures on relatively small datasets. The broad and diverse kernel pool appears to capture the essential dynamics of the sensor signals while maintaining training stability, which in turn translated into consistently strong fold-wise statistics across both benchmarks.

Second, the robustness of RP–based CNNs is noteworthy in low-concentration regimes. By visualizing recurrences of a system’s dynamic states over time, RP provides a representation that preserves subtle periodicity and non-linear behavior that are characteristic of chemical sensing processes. In contrast, GAF and MTF can be more sensitive to quantization or distributional shifts introduced during transformation, particularly when those transforms are fused early as RGB channels without prior alignment. The inconsistent performance we observed for MTF-inclusive early RGB fusion—degraded on one dataset yet strong on the other—highlights that naïvely stacking channels with disparate statistical distributions is brittle: it can unpredictably hinder or, in some cases, fortuitously enhance learning. The unambiguity of the TS2I transform is a key consideration. GAF is a theoretically bijective function that maps each signal to a unique image without information loss. In contrast, RP and MTF intentionally abstract information via thresholding or quantization to extract robust patterns. This allows different yet dynamically similar signals to produce nearly identical images, a feature that effectively preserves the core discriminative information required for classification. This behavior underscores the need for principled normalization and alignment when combining heterogeneous transforms.

Third, the limited effectiveness of transfer learning (e.g., ResNet18) points to a fundamental domain mismatch between natural images, on which these backbones are pre-trained, and the pseudo-images derived from time-series transforms. Despite dynamically adapting the first convolutional layer to accommodate multi-channel inputs, the features (edges, textures, colors) emphasized by ImageNet pretraining do not readily map onto transform-induced structures in RP/GAF/MTF. In our setting, a simpler CNN trained from scratch learned task-relevant features more effectively and, in several cases, outperformed the transferred backbone.

These observations yield practical guidance. When top accuracy with minimal tuning is the primary objective, ROCKET represents a strong default. When interpretability and qualitative examination of spatial patterns are desired, CNN-RP offers the most reliable image-based alternative in low-concentration conditions. Moreover, early fusion should be treated with caution unless cross-transform distributions are explicitly aligned.

Limitations. The present study focuses on two public datasets (GSA-LC, GSA-FM) and a fixed input resolution (e.g., 224 × 224) for compatibility across CNN backbones. Although repeated stratified K-Fold and macro-F1 were employed to mitigate class-imbalance effects and to stabilize fold-wise inference, broader datasets and alternative resolutions could further refine the conclusions and afford a fuller picture of model trade-offs.

Future Directions. Building on these results, several research avenues are promising yet prospective. (i) Stronger multi-transform fusion—including normalization schemes, learnable weighting, or attention across RP/GAF/MTF—may address the brittleness of naïve early stacking. (ii) Explainability analyses (e.g., Grad-CAM/feature visualizations, SHAP/LIME) [30,31] could illuminate decision processes in top-performing models such as ROCKET and CNN-RP, enabling principled diagnostics. (iii) A systematic efficiency study—benchmarking training/inference time, memory, and throughput—would clarify deployment feasibility; for example, exploring lower-resolution TS2I with compact, task-tailored CNNs may reduce computational and memory footprints without sacrificing accuracy. These items outline directions for future work and are not claimed by the present experiments.

## 5. Conclusions

Our benchmark provides a reproducible protocol for fair comparisons and supports three findings: (1) ROCKET achieves the best performance across both datasets; (2) RP-based CNNs are robust under low-concentration conditions; and (3) TS2I-CNNs remain competitive with modern time-series classifiers under identical manifests. These are supported by CIs and pairwise tests and are interpreted within each dataset. Collectively, they suggest that TS2I is a practical option when interpretability and cross-sensor invariance are required. No claims beyond the reported experiments are made. These conclusions offer clear, actionable guidance for model selection in this field.

This work presents a reproducible, fair benchmark for GSA classification across traditional, modern time-series, and TS2I CNN approaches, evaluated under a 20 × 5 repeated CV with a shared fold manifest. We reproduced FE-ELM and used it as a strong baseline. On both GSA-LC and GSA-FM, ROCKET achieved the best accuracy, with significant margins over TCN and MiniROCKET. Within image transforms, CNN-RP was the most robust, while CNN-GASF struggled—particularly on GSA-LC. Early RGB fusion that includes MTF significantly degraded performance relative to RGB (GADF, GASF, RP), underscoring the need for cross-transform distribution alignment before channel stacking. Late fusion (RP + GADF) was competitive but not consistently superior. Under identical transforms, ResNet18 provided no systematic advantage over SimpleCNN and was in some cases significantly worse, likely due to domain mismatch and first-layer adaptation limitations.

## Figures and Tables

**Table 1:** Hyperparameter Specifications for All Evaluated Models.

**Model**	**Parameter**	**Default Value**
FE-ELM	tau (phases)	64
	hidden (hidden neurons)	200
RVFL	hidden (hidden neurons)	200
	act (activation function)	relu’
BAGELM	n_estimators (M members)	20
	base_hidden (L neurons)	200
	ridge (Lambda λ)	1 × 10^−6^
ROCKET	n_kernels	10,000
MiniROCKET	n_features	10,000
TCN	levels (TemporalBlocks)	3
	hidden (hidden channels)	64
	epochs (max epochs)	20
	patience (early stopping)	10
	batch_size	8
	lr (learning rate)	1 × 10^−3^
**Model**	**Parameter**	**Search Space**
SVM-RBF	pca_var	[0.90, 0.95, 0.99]
	C (regularization)	1 × 10^([−4...4])^
	gamma (kernel coefficient)	1 × 10^([−4...4])^
SVM-LIN	pca_var	[0.90, 0.95, 0.99]
	C (regularization)	1 × 10^([−4...4])^
Random Forest	pca_var	[0.90, 0.95, 0.99]
	n_estimators	[100, 200, 400, 800, 1600, 3200]
KNN	pca_var	[0.90, 0.95, 0.99]
	k (neighbors)	[1, 3, 5, 7]
AdaBoost	pca_var	[0.90, 0.95, 0.99]

**Table 2:** Consolidated results for all models on GSA-FM and GSA-LC under a shared fold manifest. Values are Macro-F1 (primary); Accuracy is reported as mean ± std with 95% confidence intervals in brackets, computed from fold-wise scores over 20 × 5 repeated stratified cross-validation (*n* = 100 paired observations). Complete per-model statistics are provided in Appendix A Table A1 (GSA-FM) and Appendix A Table A2 (GSA-LC).

Family	Method	Rank (FM)	Macro-F1 (FM)	Rank (LC)	Macro-F1 (LC)
Time-Series Classifiers	ROCKET	1	0.9757	1	0.9555
	FE-ELM	2	0.9679	2	0.9407
	TCN	4	0.9503	4	0.9079
	LR	7	0.9184	12	0.8776
	SVM-LIN	9	0.9015	10	0.8829
	MiniROCKET	10	0.8997	3	0.9329
	RF	11	0.8777	16	0.8456
	SVM-RBF	12	0.8656	6	0.8984
	AdaBoost	14	0.7981	17	0.7928
	KNN	16	0.7425	15	0.8528
	ELM	17	0.7424	21	0.6392
	BAGELM	19	0.6599	13	0.8671
	RVFL	20	0.615	14	0.8602
	MLP	21	0.599	20	0.7021
Image CNN	RP	3	0.9529	8	0.8899
	GASF	5	0.9416	18	0.7529
	RGB (GADF, GASF, RP)	8	0.9024	11	0.8793
	GADF	13	0.8511	9	0.8835
	RGB (GADF, GASF, MTF)	15	0.7858	7	0.8973
	MTF	18	0.6766	19	0.7344
Fusion	RP + GADF	6	0.9343	5	0.9048

**Table 3:** Pairwise tests (GSA-LC, *n* = 100). Δ(Acc) = A − B, 95% CI, *t*, two-sided *p*, Holm within family, and $d_{z}=t/\sqrt{\left\{100\right)}$.

A	B	∆(Acc)	95% CI	*t*	*p*	*p*_Holm	$d_{z}$	Holm adj. *p* < 0.05
RP	GASF	0.12	[0.1059, 0.1341]	16.86	<1 × 10^−15^	9.80 × 10^−8^	1.686	✓
ROCKET	TCN	0.0467	[0.0341, 0.0593]	7.34	6.04 × 10^−11^	0.00836	0.734	✓
ROCKET	MiniROCKET	0.0222	[0.0103, 0.0341]	3.70	3.54 × 10^−4^	0.3821	0.370	
ROCKET	FE-ELM	0.02	[0.0078, 0.0322]	3.24	0.00163	0.4076	0.324	
RP	GADF	0.0044	[−0.0107, 0.0195]	0.60	0.7698	1	0.060	
FE-ELM	MiniROCKET	0.0022	[−0.0057, 0.0101]	0.2	0.550	1	0.040	

**Table 4:** Pairwise tests (GSA-FM, *n* = 100). Δ(Acc) = A − B, 95% CI, *t*, two-sided *p*, Holm within family, and $d_{z}=t/\sqrt{\left\{100\right)}$.

A	B	∆(Acc)	95% CI	*t*	*p*	*p*_Holm	$d_{z}$	Holm adj. *p* < 0.05
ROCKET	TCN	0.0518	[0.0312, 0.0724]	5	2.48 × 10^−6^	0.0984	0.5	
ROCKET	MiniROCKET	0.0794	[0.0612, 0.0976]	8.66	9.08 × 10^−14^	0.00229	0.866	✓
ROCKET	FE-ELM	0.0067	[−0.0054, 0.0188]	1.1	0.274	1	0.11	
CNN-RP	CNN-GADF	0.0891	[0.0619, 0.1163]	7.72	9.50 × 10^−12^	0.00598	0.772	✓
RGB (GADF, GASF, RP)	RGB (GADF, GASF, MTF)	0.0961	[0.0698, 0.1224]	7.24	9.79 × 10^−11^	<1 × 10^−10^	0.724	✓
TCN	MiniROCKET	0.0527	[0.0195, 0.0859]	3.26	0.00332	0.01993	0.326	✓
CNN-RP	CNN-GASF	0.0088	[−0.0113, 0.0289]	0.88	0.381	1	0.088	

**Table 5:** Transform and fusion ablation (*n* = 100). Within-backbone transform comparisons and Early RGB stacking; Δ(Acc) = A − B, 95% CI, *t*, Holm, *d_z_*. Lower block: Late fusion absolute mean ± std.

Dataset	A	B	∆(Acc)	95% CI	*t*	*p*	*p*_Holm	$d_{z}$	Holm adj. *p* < 0.05
GSA-FM	CNN-RP	CNN-GADF	0.089	[0.0619, 0.1163]	7.72	9.50 × 10^−12^	0.00598	0.772	✓
GSA-FM	CNN-RP	CNN-GASF	0.009	[−0.0113, 0.0289]	0.88	0.381	1	0.088	
GSA-FM	RGB (GADF, GASF, RP)	RGB (GADF, GASF, MTF)	0.096	[0.0698, 0.1224]	7.24	9.79 × 10^−11^	<1 × 10^−10^	0.724	✓
GSA-LC	CNN-RP	CNN-GASF	0.12	[0.1059, 0.1341]	16.86	<1 × 10^−15^	2.5 × 10^−8^	1.686	✓
GSA-LC	CNN-RP	CNN-GADF	0.004	[−0.0107, 0.0195]	0.6	0.55	1	0.06	

**Table 6:** Paired *t*-test vs. FE-ELM (metric: Accuracy). Negative ∆ means CNN < FE-ELM.

Dataset	Model	∆ (Mean)	*p*-Value
FM	CNN-RP	−0.0173	0.2838
FM	CNN-GASF	−0.0261	0.1743
FM	CNN-RGB (GADF, GASF, MTF)	−0.1536	4.6 × 10^−7^
FM	CNN-RGB (GADF, GASF, RP)	−0.0576	0.005
LC	CNN-RP	−0.0333	0.0699
LC	CNN-GASF	−0.1533	9.0 × 10^−8^
LC	CNN-RGB (GADF, GASF, MTF)	−0.0244	0.156
LC	CNN-RGB (GADF, GASF, RP)	−0.04	0.0648

**Table 7:** Transfer Learning vs. SimpleCNN (*n* = 100). Same-transform comparisons between ResNet18 and SimpleCNN; Δ(Acc) = A − B, 95% CI, *t*, *d_z_*, and *p*.

Dataset	Transform	A	B	∆(Acc)	95% CI	*t*	*p*	pHolm	$d_{z}$	Holm adj. *p* < 0.05
GSA-FM	RP	ResNet18	CNN	−0.013	[−0.0238, −0.0022]	−2.38	0.0192	0.0385	−0.238	✓
GSA-FM	GADF	ResNet18	CNN	−0.0118	[−0.0368, 0.0132]	−1	0.3197	0.6204	−0.1	
GSA-LC	GADF	ResNet18	CNN	−0.0244	[−0.0386, −0.0102]	−3.40	9.73 × 10^−4^	0.1019	−0.34	✓

## Data Availability

The datasets presented in this study are openly available in the UCI Machine Learning Repository. The “Gas sensor array low-concentration” dataset [25], which is further de-scribed in the research paper [1], is available at https://doi.org/10.24432/C5CK6F. The “Gas sensor array under flow modulation” dataset [26], which is further described in the research paper [27], is available at https://doi.org/10.24432/C5BG7G.

## Appendix A

**Table A1:** Results on GSA-FM. Mean accuracy ± std, 95% CI (*n* = 100), and Macro-F1 under a shared 20 × 5 CV manifest.

Family	Method	Accuracy (Mean ± Std)	95% CI	Macro-F1
Time-Series Classifiers	ROCKET	0.9721 ± 0.0480	[0.9627, 0.9815]	0.9757
	FE-ELM	0.9639 ± 0.0578	[0.9524, 0.9753]	0.9679
	TCN	0.9455 ± 0.0745	[0.9309, 0.9601]	0.9503
	LR	0.9030 ± 0.0928	[0.8846, 0.9214]	0.9184
	MiniROCKET	0.8927 ± 0.0885	[0.8754, 0.9100]	0.8997
	SVM-LIN	0.8911 ± 0.0826	[0.8747, 0.9075]	0.9015
	RF	0.8536 ± 0.0953	[0.8346, 0.8725]	0.8777
	SVM-RBF	0.8414 ± 0.1045	[0.8207, 0.8622]	0.8656
	AdaBoost	0.7616 ± 0.1162	[0.7385, 0.7846]	0.7981
	ELM	0.7100 ± 0.1730	[0.6757, 0.7443]	0.7424
	KNN	0.7058 ± 0.1228	[0.6814, 0.7301]	0.7425
	BAGELM	0.6421 ± 0.1477	[0.6132, 0.6710]	0.6599
	RVFL	0.5952 ± 0.1494	[0.5659, 0.6245]	0.615
	MLP	0.5840 ± 0.1684	[0.5506, 0.6174]	0.599
Image CNN (Time-Series → RP/GAF/MTF)	RP	0.9482 ± 0.0619	[0.9239, 0.9724]	0.9529
	GASF	0.9394 ± 0.0853	[0.9059, 0.9728]	0.9416
	RGB (GADF, GASF, RP)	0.9079 ± 0.0778	[0.8774, 0.9384]	0.9024
	GADF	0.8591 ± 0.1172	[0.8131, 0.9050]	0.8511
	RGB (GADF, GASF, MTF)	0.8118 ± 0.1003	[0.7725, 0.8511]	0.7858
	MTF	0.6979 ± 0.2059	[0.6172, 0.7786]	0.6766
Fusion	RP + GADF	0.9309 ± 0.0795	[0.8981, 0.9637]	0.9343

**Table A2:** Results on GSA-LC. Mean accuracy ± std, 95% CI (*n* = 100), and Macro-F1 under a shared 20 × 5 CV manifest.

Family	Method	Accuracy (mean ± std)	95% CI	Macro-F1
Time-Series Classifiers	ROCKET	0.9578 ± 0.0433	[0.9493, 0.9663]	0.9555
	FE-ELM	0.9406 ± 0.0531	[0.9300, 0.9511]	0.9407
	MiniROCKET	0.9356 ± 0.0524	[0.9253, 0.9459]	0.9329
	TCN	0.9111 ± 0.0556	[0.9002, 0.9220]	0.9079
	SVM-RBF	0.8978 ± 0.0655	[0.8848, 0.9108]	0.8984
	SVM-LIN	0.8833 ± 0.0629	[0.8708, 0.8958]	0.8829
	LR	0.8778 ± 0.0675	[0.8644, 0.8912]	0.8776
	BAGELM	0.8711 ± 0.0859	[0.8543, 0.8879]	0.8671
	RVFL	0.8644 ± 0.0865	[0.8474, 0.8814]	0.8602
	KNN	0.8522 ± 0.0733	[0.8377, 0.8668]	0.8528
	RF	0.8444 ± 0.0786	[0.8289, 0.8600]	0.8456
	AdaBoost	0.7928 ± 0.1071	[0.7715, 0.8140]	0.7928
	MLP	0.7056 ± 0.1364	[0.6785, 0.7326]	0.7021
	ELM	0.6394 ± 0.1087	[0.6179, 0.6610]	0.6392
Image CNN (Time-Series → RP/GAF/MTF)	RGB (GADF, GASF, MTF)	0.9022 ± 0.0627	[0.8776, 0.9268]	0.8973
	RP	0.8933 ± 0.0698	[0.8660, 0.9207]	0.8899
	GADF	0.8889 ± 0.0642	[0.8637, 0.9140]	0.8835
	RGB (GADF, GASF, RP)	0.8867 ± 0.0777	[0.8562, 0.9171]	0.8793
	GASF	0.7733 ± 0.0847	[0.7401, 0.8066]	0.7529
	MTF	0.7556 ± 0.0769	[0.7254, 0.7857]	0.7344
Fusion	RP + GADF	0.9089 ± 0.0733	[0.8786, 0.9391]	0.9048
